# Mesporous 3*C*-SiC Hollow Fibers

**DOI:** 10.1038/s41598-017-02147-8

**Published:** 2017-05-15

**Authors:** Yangwen Liu, Huilin Hou, Xinbo He, Weiyou Yang

**Affiliations:** 1 Institute for Advanced Materials and Technology, University of Science and Technology Beijing, Beijing, 100083 P.R. China; 2 Institute of Materials, Ningbo University of Technology, Ningbo City, 315016 P.R. China

## Abstract

In the present work, for the first time, we reported the exploration of mesoporous 3*C*-SiC hollow fibers via single-spinneret electrospinning of polyureasilazane (PSN) and polyvinylpyrrolidone (PVP) solution followed by high-temperature pyrolysis treatment. The resultant products were characterized by X-ray diffraction (XRD), field emission scanning electron microscopy (FESEM), high-resolution transmission electron microscopy (HRTEM) and N_2_ adsorption. The as-prepared hollow fibers with totally mesoporous walls were uniformly sized in diameter and high purity in morphology. They were composed of single-crystalline 3*C*-SiC nanoparticles with a surface area of 21.75 m^2^/g and average pore diameter of ~34 nm. The PSN concentration played a determined role on the formation of hollow fibers rather than the conventional solid counterparts, enabling their growth in a tunable manner. A possible mechanism was proposed for the formation of mesoporous SiC hollow fiber.

## Introduction

Porous silicon carbide (SiC) has attracted wide attention in many fields of energy production and environmental protection such as photocatalysts, catalystsupports, water purification, and thermal insulators, due to its superior mechanical properties, high thermal conductivity, low thermal-expansion coefficient, and good thermal-shock resistance, as well as its robust chemical stability and low electron affinity^1–5^. Up to date, many efforts have been put into the production of porous SiC nanostructures through different strategies^6–14^.

Electrospinning is a promising, low cost, and facile technique that has gained numerous and continuous interest, because of its capability and feasibility for mass production of organic/inorganic fibers with high qualities from polymer with diameters ranged from tens of nanometers to several micrometers^15–21^, offering considerable interest for many applications, such as catalysts, nanoparticle carriers in controlled release, nanofibrous membranes or filters, and electronic sensors^22–27^. By virtue of the simplicity and versatility of this technique assisted by subsequent carbothermal reduction, SiC dense/solid fibers/wires have been successfully generated^28–31^. Most recently, there are growing interest for fabrication of nanoporous SiC hollow fibers^32–34^, to enhance their properties and applications by increasing the surface areas and porosities. However, to the best of our knowledge, there are scarce works reported on the growth of hollow SiC fibers with high purity via single-spinneret electrospinning.

Herein, we report the exploration of mesoporous SiC hollow fibers via single-spinneret electrospinning of polyureasilazane (PSN) and polyvinylpyrrolidone (PVP), followed by high-temperature pyrolysis. We mainly focus on two points in current work: i) to realize the preparation of hollow SiC fibers with totally mesoporous walls; ii) to make the growth of the hollow fibers with uniform size in diameter and high purity in morphology in a tunable manner. It is promising that this work would inspire the study of mesoporous SiC hollow fibers with high surface area, which could have some potential applications to be utilized as catalyst supports and photocatalysts.

## Experimental Procedure

### Raw materials

Polyureasilazane (PSN, Ceraset, Kion Corporation, USA), Polyvinylpyrrolidone (PVP, *M*_*W*_ ≈ 30000, Qurchem, Sinopharm Chemical Reagent Co., Ltd, ShangHai, China), absolute ethyl alcohol, hydrofluoric acid (HF) and hydrochloric acid (HCl) were commercially available, which were directly used as received without further treatment.

### Preparation of mesoporous SiC hollow fibers

In a typical experimental procedure, the raw materials of 1.2 g PVP and 1.6 g PSN were firstly dissolved in 3.5 g absolute ethyl alcohol with stirring vigorously for 6 h. The resultant precursor microemulsions were transferred into a plastic syringe with a stainless steel nozzle (anode, diameter: 0.2 mm). The tip of the stainless steel nozzle was placed in front of a metal cathode (collector) with a fixed distance of 20 cm between the nozzle and the collector. An electrical potential of 16 kV was applied for electrospinning precursor fibers. The as-spun polymer fibers were located in a petri dish, which were dried in an oven at 80 °C for 4 h. Then the as-spun PVP/PSN fibers were cured at 200 °C for 2 h in air with a heating rate of 10 °C/min from room temperature (RT) to 100 °C, and then up to the desired temperature of 200 °C at 1 °C/min. Subsequently, the PVP/PSN fibers were located in an Al_2_O_3_ crucible to be pyrolyzed in a graphite-heater furnace under flowing Ar (100 sccm, 99.9%, 0.1 MPa). The pyrolysis procedure was heated up to the desired temperature of 1400 °C with a heating rate of 10 °C/min, and maintained there for 1 h, After the furnace was cooled down to RT, the as-prepared product was collected. The raw product was purified by air calcination at 700 °C for 4 h to eliminate the redundant carbons. Finally, the SiO_2_ layer existed around the surface of SiC fibers were removed by treatment with the mixed acid for 12 h, which are composed by 10 vol% HF + 10 vol% HCl + 80 vol% H_2_O. The sample was then leached with distilled water until the pH maintained to ~7. For comparison, five solutions were prepared with different PSN contents, as shown in Table 1. The obtained products were referred to Sample A–E, respectively.

**Table 1:** Compositions of five solutions used for electrospinning polymer precursor fibers and corresponding BET specific surface areas of pyrolyzed products.

Sample	PVP (g)	PSN (g)	Alcohol (g)	PSN (wt.%)	S_BET_ (m^2^/g)
A	1.2	0.8	3.5	14.5	16.57
B	1.2	1.0	3.5	17.5	20.42
C	1.2	1.2	3.5	20.3	15.45
D	1.2	1.4	3.5	23	20.34
E	1.2	1.6	3.5	25.4	21.75

### Structural characterization

The obtained products were characterized with X-ray powder diffraction (XRD, D8 Advance, Bruker, Germany) with Cu Kα radiation (λ = 1.5406 Å), field emission scanning electron microscopy (FESEM, S-4800, Hitachi, Japan), and high-resolution transmission electron microscopy (HRTEM, JEM-2010F, JEOL, Japan) equipped with energy dispersive X-ray spectroscopy (EDX, Quantax-STEM, Bruker, Germany). The porous properties of the as-prepared mesoporous fibers were characterized using N2 adsorption at −195.8 °C on a specific surface area and porosity analyzer (ASAP 2020HD88, Micromeritics, USA).

## Results and Discussion

**Figure 1:**
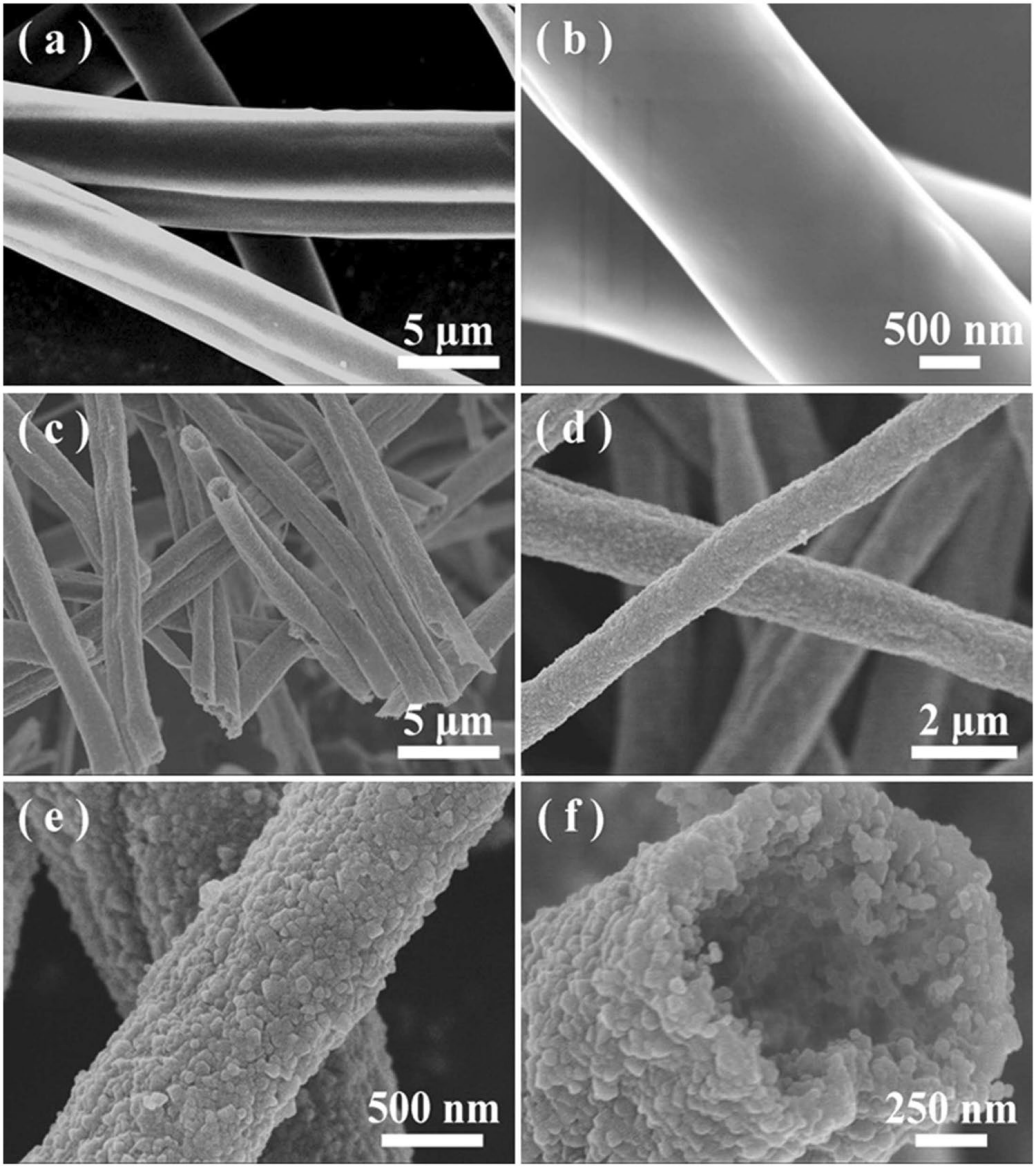
(**a** and **b**) Typical SEM images of the as-spun PVP/PSN fibers of Sample E. (**c** and **d**) Typical SEM images of the pyrolyzed products under different magnifications. (**e**) Representative SEM images of the surface structures of the as-fabricated mesoporous SiC hollow fibers. (**f**) A representative SEM image showing the fracture surface of the mesoporous hollow fibers.

SEM was firstly employed to study the morphology and microstructure of the precursor fibers of Sample E. The resultant precursor fibers (Fig. 1(a,b)) are continuous with the diameters in the range of 2~4.5 μm with a smooth surface and typical length up to several hundred of millimeters. Figure 1(c–f) are the typical SEM images of the corresponding pyrolyzed products under different magnifications and views. It seems that the long continuous precursor fibers have been converted into hollow structures with the diameters reduced to 1~2 μm. The resultant fibers are uniform in diameter with a high purity in morphology. Figure 1(e,f) show the interior and external surface of the hollow fibers under different magnifications, respectively, which disclose that numerous pores exist on the surface and walls, representing that the as-prepared fibers are totally mesoporous. The formation of hollow and mesoporous structures is mainly attributed to the decomposition and removing of organics within the as-spun polymeric fibers.

**Figure 2:**
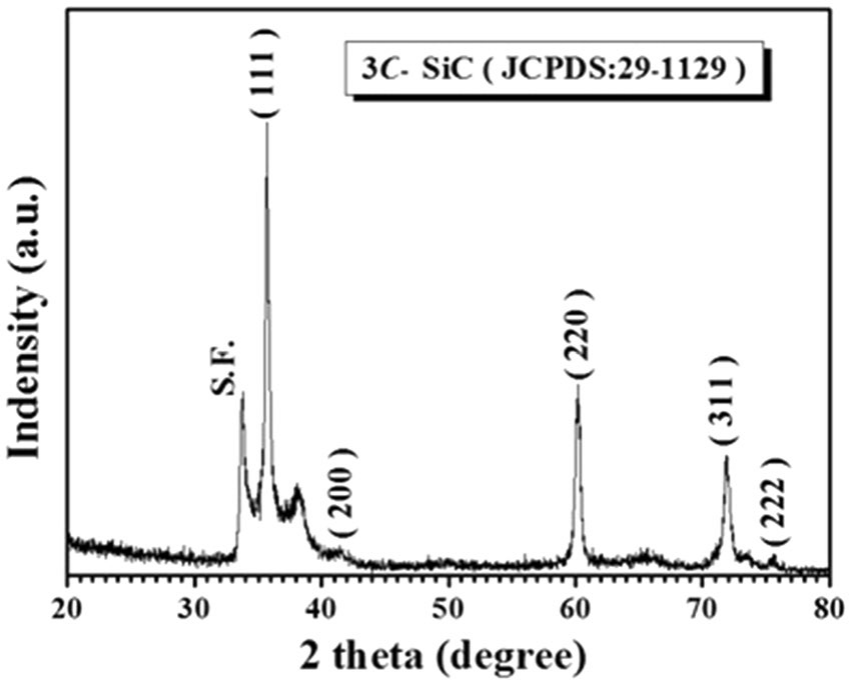
Representative XRD pattern recorded from Sample E after pyrolyzation at 1400 °C for 1 h.

Figure 2 presents the typical XRD pattern of the resultant hollow fibers of Sample E. The peak sets well match the phase of 3*C*-SiC (JCPDS, No. 29–1129), which can be attributed to the fact that SiC is more stable than Si_3_N_4_, according to the Si–C–N ternary diagram at 1400 °C under an Ar atmosphere^35^ (PSN and PVP composed mainly of Si, C, N, and H elements). The strong and sharp diffraction peaks indicate that the products are highly crystalline. A small peak (2θ = 33.8°) marked with S.F. can be ascribed to the stacking faults within the 3*C*-SiC structure^36^. The stacking faults might reassemble the structures of other polytype phases of SiC such as α-SiC within the 3*C*-SiC matrix^37, 38^. Accordingly, the minor peak (2θ = 38.2°) might be assigned to the diffraction of the (103) crystal plane of α-SiC.

**Figure 3:**
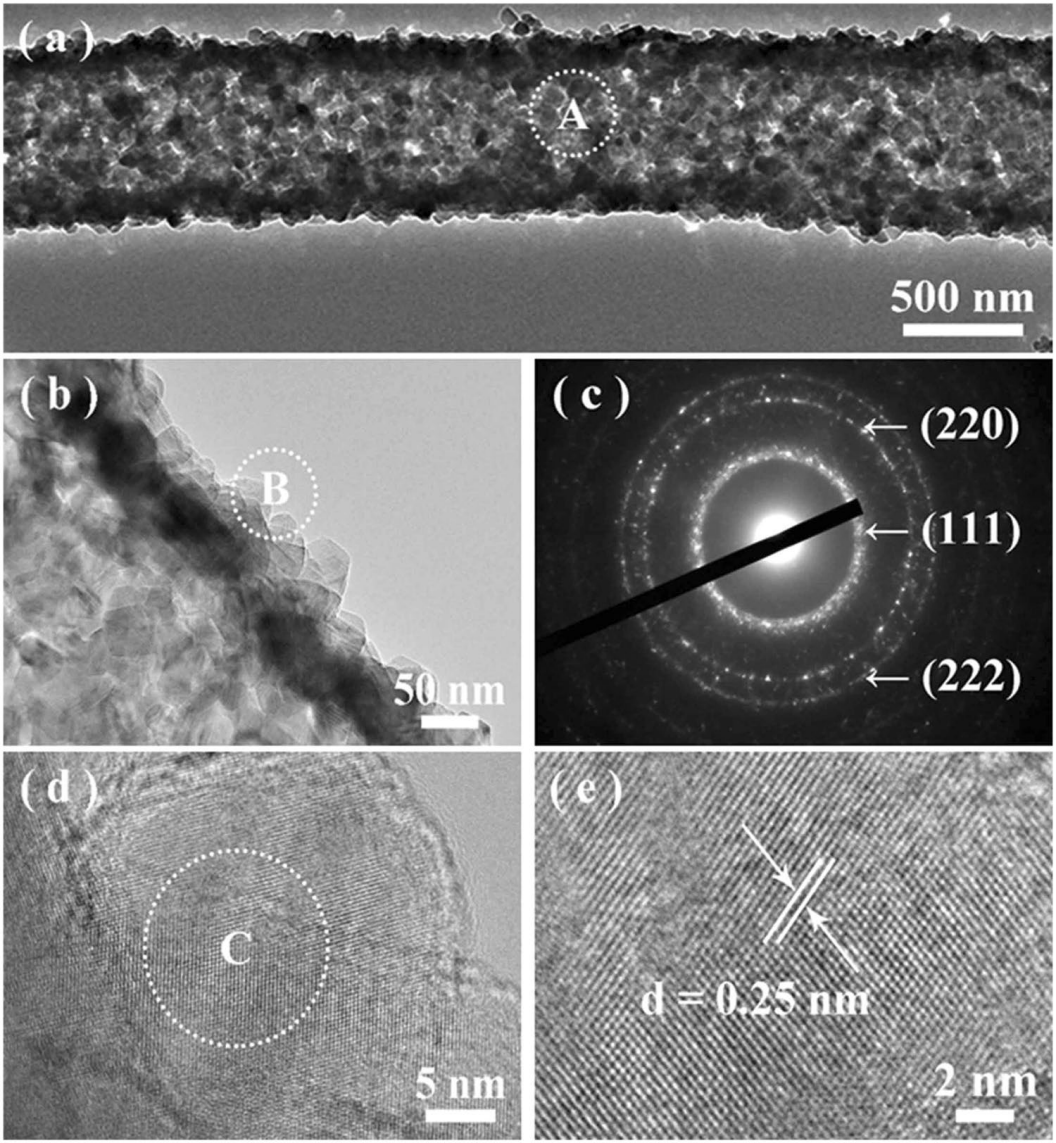
(**a** and **b**) Typical TEM images of a single follow SiC fiber from Sample E under different magnifications. (**c**) A typical SAED pattern recorded from the marked area of A in part a. (**d**) Typical HRTEM image obtained from a single nanoparticle recorded from the marked area of B in part b. (**e**) An enlarged HRTEM image recorded from the marked area of C in part d.

TEM was further used to investigate the morphology and structural details of the hollow fiber, as shown in Fig. 3(a). In agreement with the SEM observations, the inner tunnel is clearly observed by the sharp contrast between SiC mesoporous wall and the hollow interior. The closer observation (Fig. 3b) presents that the fibers typically consist of irregular plate-like nanoparticles. The chemical compositions are identified by EDX under TEM recorded from a single fiber, suggesting that they mainly consist of Si and C, with a little amount of Al and O elements (the Cu signals are from the TEM copper grids) (Figure S1 in Supporting Information). The atomic ratio of Si to C, within the experimental limit, is close to 1:1, suggesting the fibers are SiC. The detected Al and O elements should be attributed to the introduced impurities from the used Al_2_O_3_ crucible for the high-temperature pyrolysis treatment and the absorbed oxygen when the fibers were exposed in air, respectively. Figure 3c presents a typical selective area electron diffraction (SAED) pattern recorded from the whole fiber body (marked area of A in Fig. 3a), suggesting its polycrystalline nature with a high crystallinity. The diffraction spot rings could be sequentially indexed to be the (111), (220), and (222) crystal planes of 3*C*-SiC (JCPDS, No. 29–1129), further confirming that the resultant hollow fibers are of pure -SiC. Figure 3d and e present the representative HRTEM images recorded from a single particle, which are obtained from the marked area of B in Fig. 3b and c, respectively, disclosing the single-crystalline nature of the nanoparticles. That is to say, the as-fabricated hollow SiC fibers are composed of single-crystalline nanoparticles.

**Figure 4:**
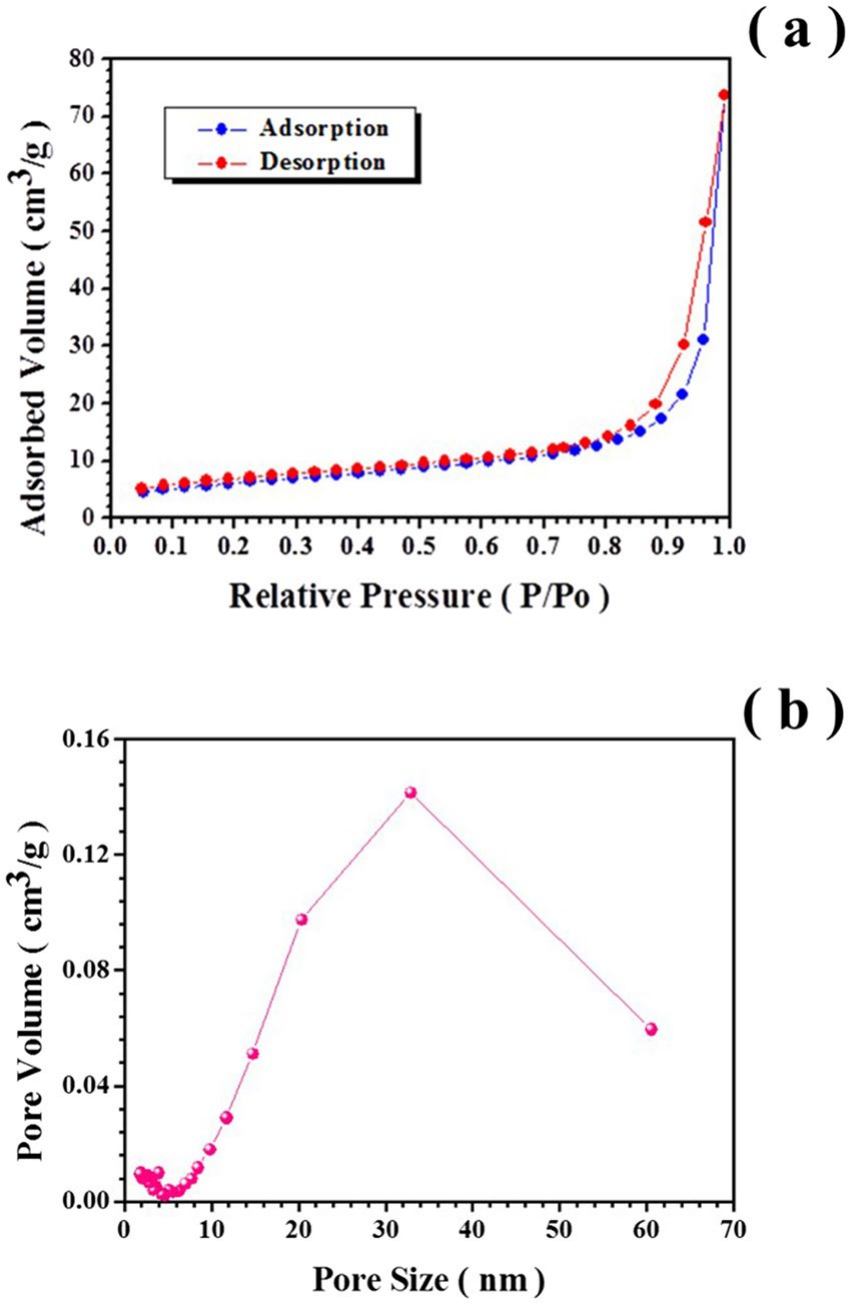
(**a**) Nitrogen adsorption-desorption isotherm curve of Sample E. (**b**) Pore size distribution curve of Sample E.

The nitrogen adsorption measurements as shown in Fig. 4(a) reveal that the as-fabricated products exhibit the type IV isotherm behavior with H3 hysteresis, implying that the obtained hollow fibers are mesoporous with a BET surface area of 21.75 m^2^/g. According to the Barrett–Joyner–Halenda (BJH) pore size distribution analysis determined from the adsorption branches (Fig. 4(b)), the average BJH pore diameter is ~34 nm.

**Figure 5:**
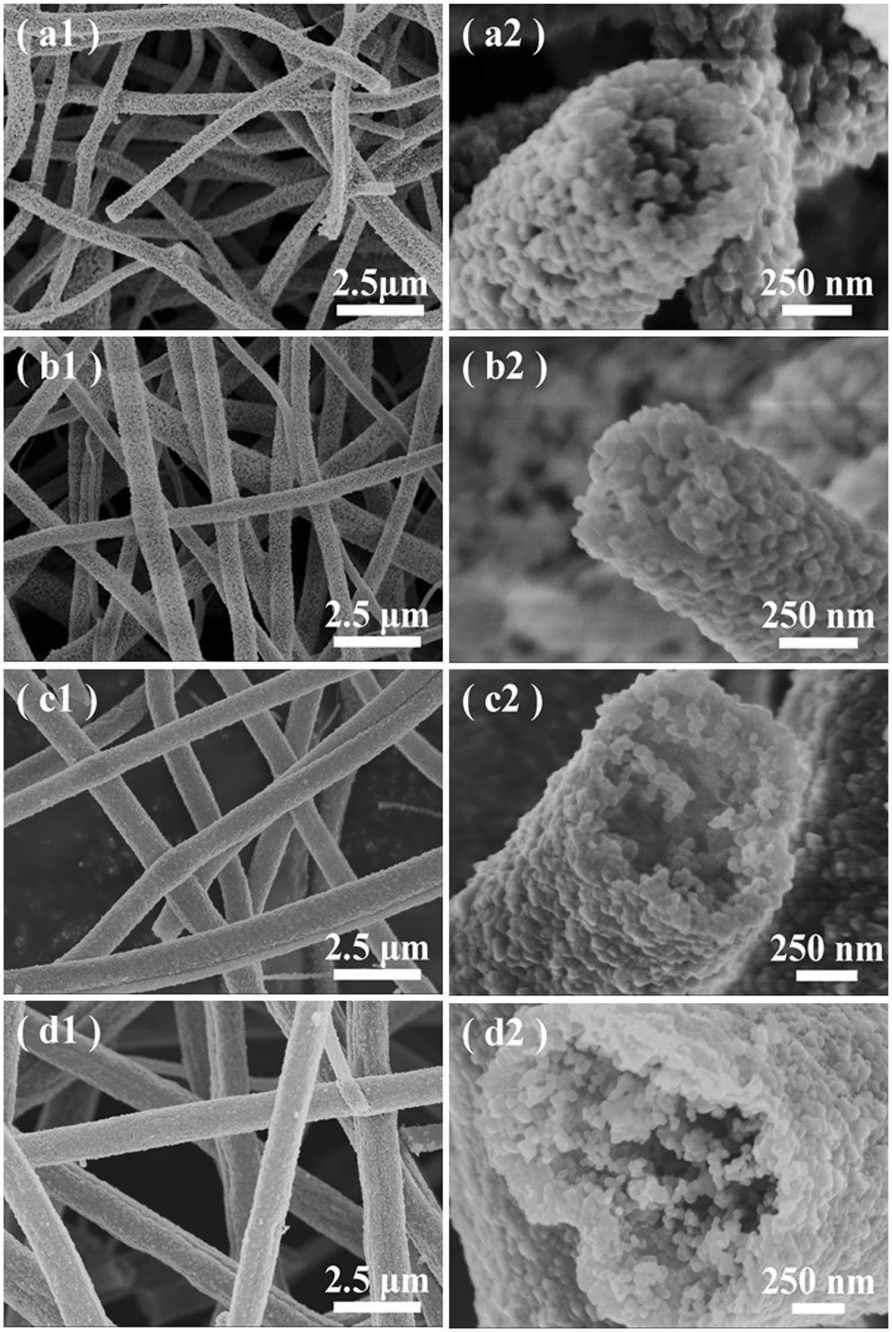
Typical SEM images of pyrolyzed products of Sample A (a1–a2), Sample B (b1–b2), Sample C (c1–c2), and Sample D (d1–d2) under different magnifications.

To disclose the growth of hollow SiC fibers, another four comparative experiments are carried out by adjusting the PSN contents and keeping the PVP and alcohol constant (Table 1). The experimental results suggest that all the pyrolyzed products of Sample A–E are of 3*C*-SiC phase (Fig. 2, Figure S2 in Supporting Information), implying that the change of the PSN contents has little influence on the phase formation of the final products. The BET specific areas of the hollow samples show a little change with the variation of the introduced PSN contents (Table 1, Figure S3 in Supporting Information). With a low concentration of PSN introduced in the raw materials (*i.e*., Sample A and B), the conventional solid fibers would be formed (Fig. 5(a1,a2 and b1,b2)). Once the PSN contents is up to ~20 wt.% (*i.e*., Sample C), the fibers with hollow interior seem to be formed. The growth of the hollow fibers could be accomplished with more and more PSN introduced into the raw materials (*i.e*., Sample D and E, Figure 5c1,c1 and d1,d2). The diameters of the as-prepared fibers are increased with the more PSN concentrations, which could be mainly attributed to various viscosities and dielectric constant, since a larger diameter of the polymer fibers would be spun by increasing the viscosity of the solution (Table 1, Figure S4 in Supporting Information). It suggests that the PSN concentration plays a determined role on the formation of the hollow SiC fibers, enabling their growth in a controlled manner. In our case, the PSN concentration high up to 20 wt.% is necessary for the formation of hollow SiC nanofibers.

Based on the experimental results as mentioned above, the growth of mesoporous SiC hollow fibers is proposed as: i) The formation of the hollow interior. In regard to the Sample A-E, the only difference is the various PSN contents introduced within the raw materials. As compared to the initial diameter of the as-spun polymeric fibers, those of the pyrolyzed counterparts are often obviously shrunk, due to the elimination of the organics such as PVP within the polymeric fibers caused by the high-temperature treatment. As shown in Figs 1 and 5, with more PSN introduced, the diameter of the resultant SiC fibers is larger. This implies that the outlayer of inorganic SiC for the pyrolyzed fibers could be prior formed with a high enough PSN introduced to the raw materials (*i.e*., with high enough Si contents from the PSN for the formation of SiC). The formed SiC outlayer might hinder the shrinkage of the fibers. Subsequently, the further elimination of the organics from the interior part of the polymeric fiber causes the formation of the hollowed interior; ii) The formation of the thoroughly mesoporous walls. It is mainly attributed to the selective evaporation/calcination mechanism^23, 30^, due to the two distinctively different thermal properties between PSN and PVP. It is known that the PSN mainly contains of Si, C, and N elements with a small amount of O (Figure S5 in Supporting Information), which would be converted into amorphous SiCN solids and can be thermally stable up to 1000 °C^39, 40^. However, the PVP is mainly composed of C, N, H, and O elements (Figure S5 in Supporting Information), which would be completely decomposed into vapor phases such as NH_3_, CH_4_, and CO_2_ when heated up to ~500 °C^41, 42^. This is verified by the analysis of the thermal behaviors of as-spun PSN/PVP fibers from Sample E (Figure S6 in Supporting Information). Once subjected to be calcinated at high temperature, the PSN would be converted into inorganic SiC for constructing the walls of the hollow fibers, and the PVP would be completely decomposed into gas phases, making the creation of mesopores throughout the body of the fiber walls.

## Conclusions

In summary, we have demonstrated the exploration of mesoporous SiC hollow fibers via single-spinneret electrospinning technique with the subsequent high-temperature pyrolysis treatment. The hollow SiC fibers with totally mesoporous walls are uniformly sized in diameter and high purity in morphology. They are composed of single-crystalline 3*C*-SiC nanoparticles with a surface area of 21.75 m^2^/g and average pore diameter of ~34 nm. It is found that the PSN concentration within the raw materials played a determined role on the formation of hollow fibers, making their growth in a tunable manner. The growth of the mesoporous SiC hollow fibers could be mainly attributed to the selective evaporation/calcination of PSN and PVP. The as-prepared mesoporous hollow fibers could have some potential applications in photocatalysts, catalyst supports and supercapacitor, owing to their light weight, high surface area as well as their hollow and mesoporous characteristics.

## Electronic supplementary material

Mesporous 3C-SiC Hollow Fibers
