# Supplementary material for: Mesporous 3C-SiC Hollow Fibers
Source: Sci Rep. 2017 May 15;7:1893. doi: 10.1038/s41598-017-02147-8 (PMC5432512; doi:10.1038/s41598-017-02147-8)
Supplement: Supplementary file 1 — Mesporous 3C-SiC Hollow Fibers [file 41598_2017_2147_MOESM1_ESM.doc]

***Supporting Information***

**Mesporous 3*C*-SiC Hollow Fibers**

*Yangwen Liu^1,2^, Huilin Hou^2^, Xinbo He^1,*^ and Weiyou Yang2,[[1]](#footnote-2)**

^1^ Institute for Advanced Materials and Technology, University of Science and Technology Beijing, Beijing, 100083, PR China

^2^ Institute of Materials, Ningbo University of Technology, Ningbo City, 315016, P.R. China.

***Corresponding author E-mails:*** xb_he@163.com (X. He)

[weiyouyang@tsinghua.org.cn](mailto:weiyouyang@tsinghua.org.cn) (W. Yang)

***Tel:*** +86-574-87080966

***Fax:*** +86-574-87081221


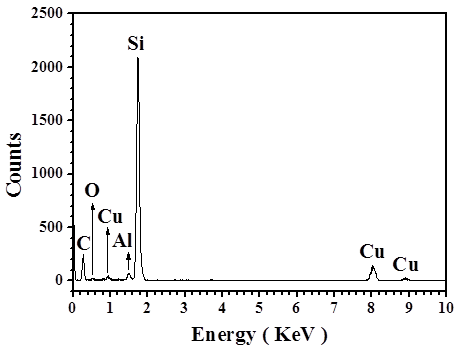


**Figure S1.** A typical EDX spectrum of the resultant SiC fibers.


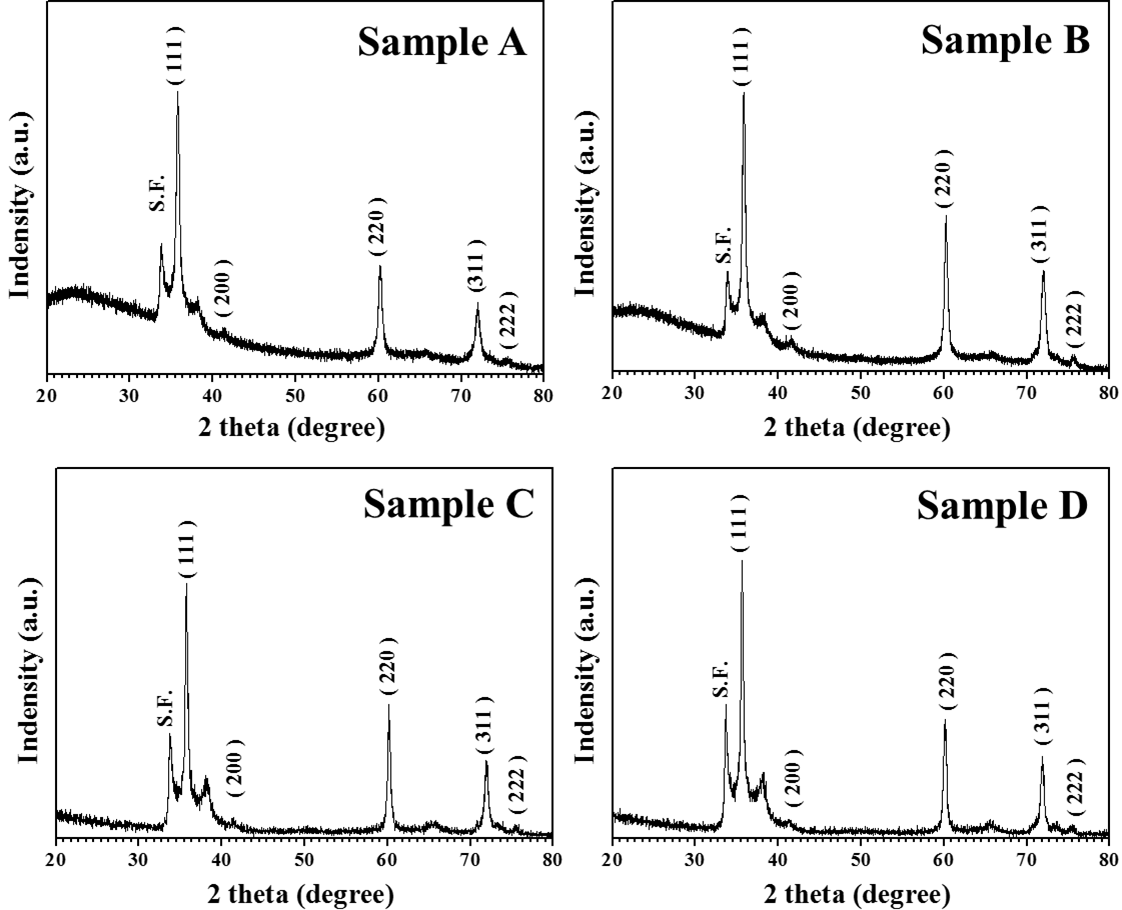


**Figure S2.** Respective XRD patterns recorded from Sample A, B, C and D after pyrolyzed at 1400 °C for 1 h, respectively.


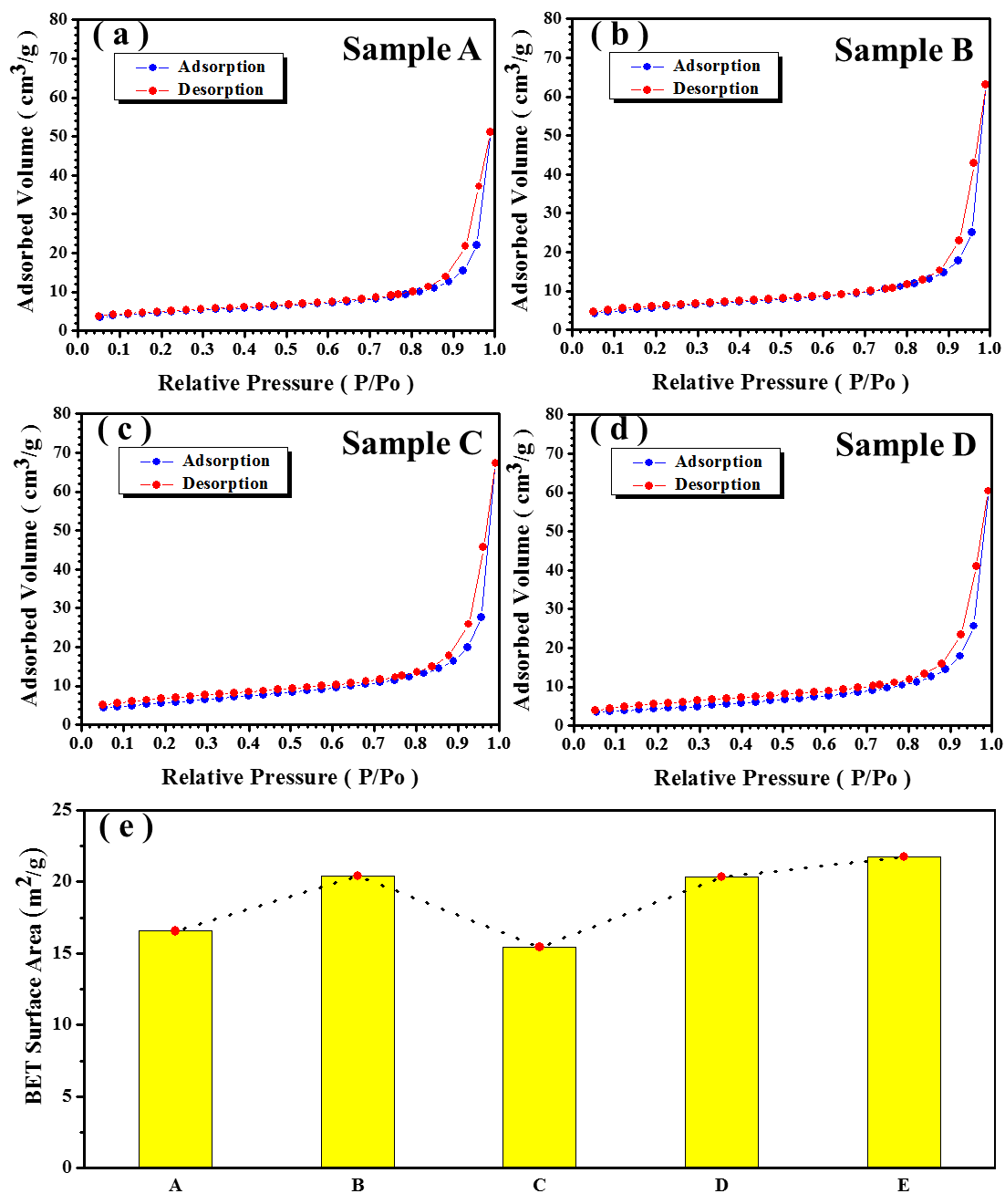


**Figure S3.** （a-d） Respective Nitrogen adsorption-desorption isotherm curves from Sample A, B, C and D after pyrolyzed at 1400 °C for 1 h, respectively. (e) The BET surface areas of sample A, B, C, D and E after pyrolyzed at 1400 °C for 1 h.


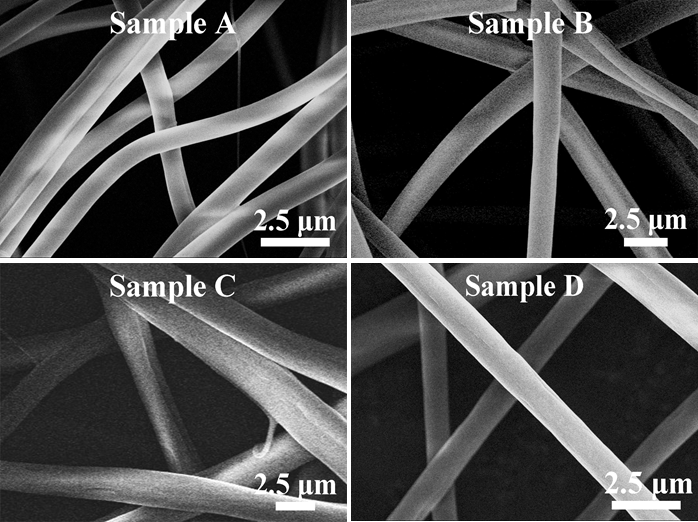


**Figure S4.** Typical SEM images of the electrospun PVP/PSN polymer precursor fibers from Sample A, B, C and D, respectively.


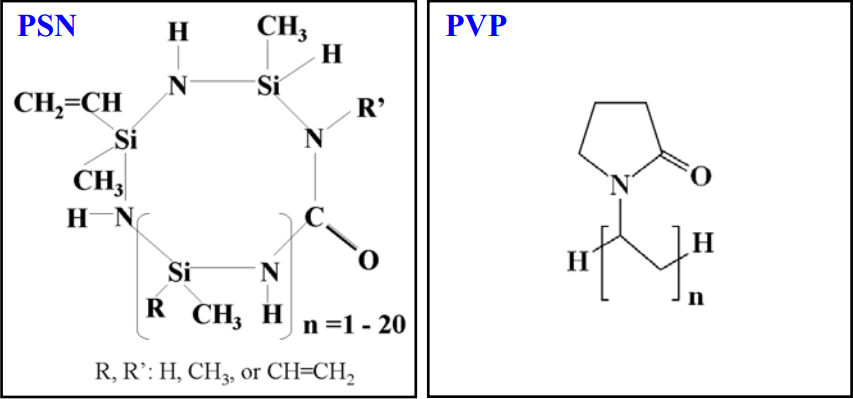


**Figure S5.** Molecular structures of PSN and PVP.


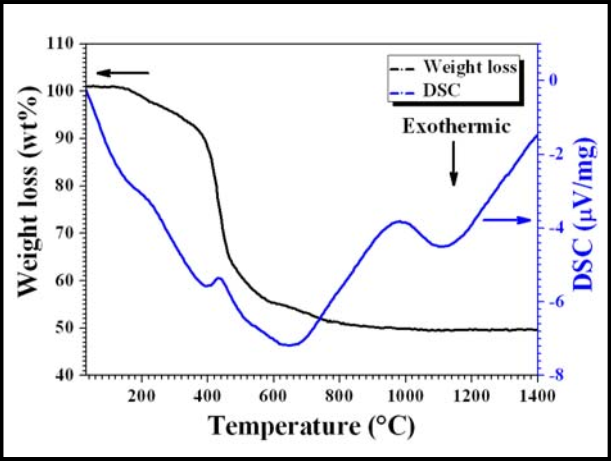


**Figure S6.** TGA-DSC curves showing the thermal behaviors of polymeric precursor fibers.（Data from J. Crystal Growth & Design, 2012, 12(1): 536-539）

1. ^^ Corresponding authors. E-mails: xb_he@163.com (X.He) and [weiyouyang@tsinghua.org.cn](mailto:weiyouyang@tsinghua.org.cn) (W. Yang)

   Tel: +86-574-87080966, Fax: +86-574-87081221. [↑](#footnote-ref-2)
